# Diacetylenic lipids in the design of stable lipopolymers able to complex and protect plasmid DNA

**DOI:** 10.1371/journal.pone.0186194

**Published:** 2017-10-11

**Authors:** C. Facundo Temprana, M. Jimena Prieto, Daniela E. Igartúa, A. Lis Femia, M. Silvia Amor, Silvia del Valle Alonso

**Affiliations:** 1 Laboratorio de Biomembranas (LBM), Departamento de Ciencia y Tecnología, Universidad Nacional de Quilmes, Bernal, Argentina; 2 Grupo vinculado GBEyB, IMBICE, CICPBA, CCT, La Plata – CONICET; Helsingin Yliopisto, FINLAND

## Abstract

Different viral and non-viral vectors have been designed to allow the delivery of nucleic acids in gene therapy. In general, non-viral vectors have been associated with increased safety for *in vivo* use; however, issues regarding their efficacy, toxicity and stability continue to drive further research. Thus, the aim of this study was to evaluate the potential use of the polymerizable diacetylenic lipid 1,2-bis(10,12-tricosadiynoyl)-sn-glycero-3-phosphocholine (DC_8,9_PC) as a strategy to formulate stable cationic lipopolymers in the delivery and protection of plasmid DNA. Cationic lipopolymers were prepared following two different methodologies by using DC_8,9_PC, 1,2-dimyristoyl-sn-glycero-3-phosphocholine (DMPC), and the cationic lipids (CL) 1,2-dioleoyl-3-trimethylammonium-propane (DOTAP), stearylamine (SA), and myristoylcholine chloride (MCL), in a molar ratio of 1:1:0.2 (DMPC:DC_8,9_PC:CL). The copolymerization methodology allowed obtaining cationic lipopolymers which were smaller in size than those obtained by the cationic addition methodology although both techniques presented high size stability over a 166-day incubation period at 4°C. Cationic lipopolymers containing DOTAP or MCL were more efficient in complexing DNA than those containing SA. Moreover, lipopolymers containing DOTAP were found to form highly stable complexes with DNA, able to resist serum DNAses degradation. Furthermore, neither of the cationic lipopolymers (with or without DNA) induced red blood cell hemolysis, although metabolic activity determined on the L-929 and Vero cell lines was found to be dependent on the cell line, the formulation and the presence of DNA. The high stability and DNA protection capacity as well as the reduced toxicity determined for the cationic lipopolymer containing DOTAP highlight the potential advantage of using lipopolymers when designing novel non-viral carrier systems for use in *in vivo* gene therapy. Thus, this work represents the first steps toward developing a cationic lipopolymer-based gene delivery system using polymerizable and cationic lipids.

## Introduction

Non-viral and viral vectors are used in gene therapy. This therapy has evolved as a strategy to treat different acquired or inherited diseases in which a gene defect is responsible for the pathological condition. Gene therapy allows “repairing” the defective gene by the delivery of an exogenous right copy of it [1–6]. Although the principles behind gene therapy are simple, the delivery of such genes is still a challenge. Different viral and non-viral vectors have been designed to allow the delivery of such genes, but the ideal vector has not been found yet [7–12].

In the last decades, liposomes have been extensively studied as drug delivery systems [13]. These kinds of systems are very useful since they can protect and carry both lipophobic and/or lipophilic drugs, they can be targeted to different sites in the organism, and they can reduce drug side effects by lowering the amount of “free” drug in plasma [14–21]. Since liposomes can also interact with DNA and protect it from enzymatic degradation, special interest has arisen in these systems when designing a potential non-viral DNA carrier [6, 14, 22–32]. However, the stability of these delivery systems is still an important issue to be improved [7, 11, 33].

It has been discussed that polymerizable lipids can be used to enhance membrane stability, both physically and chemically, after polymerization [34–44]. In line with this idea, formulating liposomes containing polymerizable diacetylenic lipids would allow obtaining higher system stability after polymerization, and thus confer higher stability to the lipopolymer/DNA complex. It has been previously reported that polymeric liposomes have little interaction with DNA [23]. Recently, it has been shown that the mixture of the polymerizable diacetylenic lipid 1,2-bis(10,12-tricosadiynoyl)-sn-glycero-3-phosphocholine (DC_8,9_PC) and the lipid 1,2-dimyristoyl-sn-glycero-3-phosphocholine (DMPC) (1:1) itself has the ability to transfect cells *in vivo* in mice [45] after being administered intratracheally. These results give the first indication that lipopolymers formulated with the diacetylenic lipid DC_8,9_PC might be used for *in vivo* DNA delivery. However, to our knowledge, there are no reports dealing with the optimization of delivery systems containing polymerizable lipids to increase DNA interaction and protection. Thus, the main aim of this study was to evaluate the potentiality of using polymerizable lipids in the design of new non-viral vectors for gene delivery. To this end, we evaluated the possibility to improve the DNA interaction of the mixture between the polymerizable diacetylenic lipid DC_8,9_PC and the lipid DMPC, by analyzing an appropriate methodology to incorporate 1,2-dioleoyl-3-trimethylammonium-propane (DOTAP), stearylamine (SA), or myristoylcholine chloride (MCL), as cationic lipids (CL) into the lipopolymer, and determining the amount of cationic lipopolymer needed to associate plasmid DNA. To study the interaction of diacetylenic cationic lipopolymers with plasmid DNA, flow cytometry was used in combination with the gel retardation assay. We also studied the effect of plasmid size, the Z-potential, the effect of different media in the cationic lipopolymer/DNA interaction, the DNA protection from serum DNAses, and the cytotoxicity of the different systems. All these characterizations revealed an adequate methodology to obtain cationic lipopolymer and gave an insight of the potentiality of this novel system as non-viral vectors for gene delivery.

## Materials and methods

### Materials

The polymerizable lipid DC_8,9_PC was from Avanti Polar Lipids Inc. and the phospholipid DMPC from Lipoid GmbH. DOTAP and MCL were from Toronto Research Chemicals Inc. and SA from Fluka. 3-(4,5-dimethyl-2-thiazolyl)-2,5-diphenyl tetrazolium bromide (MTT) was from USB Corporation. SYBR^®^ Green I was from Molecular Probes, cell culture MEM/EBSS NEAA modified medium was from HyClone and antibiotic-antimycotic was from Gibco. All other reagents were of analytical grade and used without further purification. Plasmids pCH110 and pDsRed2-N1 were a generous gift from Dr. Víctor Romanowski from Instituto de Bioquímica y Biología Molecular (IBBM), Universidad Nacional de La Plata (UNLP), Consejo Nacional de Investigaciones Científicas y Técnicas (CONICET), Argentina.

### Liposome preparation

Liposomes were prepared according to Bangham *et al*. (1965) [46]. Briefly, lipids were dissolved in chloroform, and the solvent was evaporated until a thin dry film was obtained. The film was flushed with nitrogen and then suspended in distilled water, in general to a final 5 mM lipid concentration. In a typical formulation, lipids were used in a 1:1 molar ratio for DMPC:DC_8,9_PC, or in a 1:1:0.2 molar ratio for DMPC:DC_8,9_PC:CL. Then, the suspension was extruded at 50°C fifteen times through 0.2-μm-pore polycarbonate membranes, using a Mini Extruder from Avanti Polar Lipids. Two different methodologies were used to incorporate the CL into the DMPC:DC_8,9_PC (1:1) formulation. One methodology (from now on called “copolymerization”) included the CL mixed with the other two lipids (DMPC and DC_8,9_PC) being dissolved in chloroform, as stated before. The other methodology (from now on called “cationic addition”), involved: 1) the preparation of the DMPC:DC_8,9_PC (1:1) lipopolymer (see *Extruded Vesicle Polymerization*); 2) lyophilization from a frozen suspension (-80°C overnight) in a Freezone 4.5, LABCONCO lyophilizer (Kansas City, MO, USA), pre-cooled at -50°C maintaining the lyophilization process pressure within the range of 33x10^-3^ to 65x10^-3^ mbar for 24 h; 3) the dissolution of the lyophilized powder in chloroform; 4) the addition of a CL solution (prepared in chloroform) to obtain the final DMPC:DC_8,9_PC:CL 1:1:0.2 molar ratio; 5) the evaporation of the solvent until a thin dry film was obtained; 6) and finally, the film suspension in distilled water to obtain a final 5 mM lipid concentration.

### Extruded vesicle polymerization

Diacetylenic vesicles were polymerized under 254 nm UV light (20 cycles of 360 mJ/cm^2^ each), using a UV-Stratalinker 1800, Stratagene^®^. The temperature was maintained at 4°C for 5 min in between cycles. Spectra were recorded with a Nanodrop ND-100 spectrophotometer (Thermo Scientific), between 400 and 700 nm at room temperature [34, 47–49].

### Size measurements

The size of both the vesicles and complexes was determined at 25°C by measuring the autocorrelation function at a 90° scattering angle in a 90 Plus/Bi-MAS Particle Size Analyzer (Brookhaven Instruments Corporation), with a light source of 632.8 nm and a 10-mW laser. Each result is the average of three measurements and the effective diameters are reported as number-based diameters. The size stability of the cationic lipopolymers was determined for samples that were kept at 4°C until analyzed. Samples were lightly vortexed before measurements that were carried out on days 1 and 166 after sample preparation. Data acquisition and analysis were conducted using the software package (Brookhaven Instruments 90Plus Particle Sizing Software) supplied by the manufacturer.

### Plasmid DNA

Plasmid DNAs pCH110 (7128 bp) and pDsRed2-N1 (4692 bp) were purified from *Escherichia coli* hosts, using a Wizard^®^ Plus Midipreps DNA purification System from Promega, following the instructions provided by the manufacturer. DNA concentration (absorbance at 260 nm) was determined with a Nanodrop ND-100 spectrophotometer. The DNA used in this work presented absorbance at a 260/280 nm ratio higher than 1.75.

### Gel retardation assay

The cationic lipopolymers and DNA were mixed in a final volume of 20 μL of sterile distilled water and then incubated at 37°C for 30 min. Then, 8 μL of gel loading buffer (10 mM Tris-HCl pH 8, 2 mM EDTA, 0.01% w/v Orange G and 10% v/v glycerol) was added before loading the sample onto an 0.8% w/v agarose gel containing ethidium bromide in a 0.1 μg/mL final concentration. Electrophoresis was performed for 60 min in a Max horizon sub w/cast kit comb apparatus (Amersham Biosciences), maintaining the voltage constant at 105 V with an EC 105 power supply from E-C Apparatus Corporation, using TAE 1X (40 mM Tris, 20 mM acetic acid, 1 mM EDTA, pH 8.0) as running buffer. Gels were viewed under a UV transilluminator and images were captured with a Kodak camera using the Kodak Digital Science 1D software. To evaluate the amount of cationic lipopolymer necessary to complex a specific amount of plasmid DNA, as well as the effect of plasmid size, different cationic lipopolymer/plasmid DNA ratios (expressed as mol of lipids: mol of base pairs) were evaluated (0:1, 1:1, 2:1, 3:1, 4:1, 5:1, 6:1, 8:1, 10:1, 12:1, 14:1, 16:1, 24:1, 30:1, 36:1, 42:1). These mol of lipids: mol of base pairs ratios are equivalent to amine: phosphate ratios of 0, 0.045, 0.091, 0.136, 0.182, 0.227, 0.273, 0.364, 0.455, 0.546, 0.637, 0.728, 1.092, 1.365, 1.638, and 1.911, respectively [50–52]. In all the ratios assayed, 1 μg of plasmid DNA was used. For the analysis, in each gel, a reference sample of 1 μg of “free” plasmid was included and the densitometry value obtained in the quantification of the corresponding negatively supercoiled plasmid band was set as the 100% plasmid amount used in the experiment. Then, the values obtained for this negatively supercoiled plasmid band present in the other samples (incubated with lipopolymers) within the same gel were referred to as control to calculate the percentage of complexed plasmid, with the Kodak Digital Science 1D software. Results are the mean of at least three independent experiments and are expressed as association percentage (100% minus the percentage of non-complexed plasmid).

### Z-potential

The Z-potentials of the cationic lipopolymers were determined for a final 50 μM lipid concentration in distilled water at 25°C by phase-analysis light scattering in a Nanosizer (ZEN 3600; Malvern Instruments, Malvern, UK) [53]. Each result is the average of three independent measurements.

### Effect of different incubation media in the cationic lipopolymer/DNA interaction

The cationic lipopolymer/DNA complexes were formed in water, PBS (pH 7.4, 10 mM) or MEM (HyClone, prepared as specified by the manufacturer). Complexes were prepared as stated previously, at a 16:1 mol of lipids: mol of base pairs ratio, in the different media with and without FBS (10% final concentration) and then incubated for additional 10 min with or without 10% or 50% FBS. Complexes were analyzed through the *Gel Retardation Assay* as described above.

### Serum nucleases digestion assay

The cationic lipopolymer/DNA complexes were prepared in water, PBS and MEM as explained in the *Gel Retardation Assay* section, with a 16:1 mol of lipids: mol of base pairs ratio. After the 30-min incubation at 37°C used to induce the formation of the complexes, FBS was added to a final 50% v/v concentration and samples were incubated for additional 24 h at 37°C before agarose gel electrophoresis analysis, which was performed as described in the *Gel Retardation Assay* section [54].

### Flow cytometry

The formation of complexes between the cationic lipopolymers and the pDsRed plasmid was studied through flow cytometry. For each detected event, we recorded the FSC-H and SSC-H values, as well as the fluorescence intensity in the FL1 (bandpass 530/30 nm) and FL2 (bandpass 585/42 nm) detectors after excitation with a 488 nm wavelength from an argon-ion laser in a FACS Calibur cytometer (BD). Data acquisition and analysis were performed using the Cell Quest Pro software supplied by BD.

### Hemolysis

The study was conducted in accordance with the principles of the Declaration of Helsinki and was approved by the Ethics Committee of National University of Quilmes (Buenos Aires, Argentina; ethics CE-UNQ No 2/2014). The participant (healthy donor) provided a written informed consent to the experimental protocol before his/her study-participation.

Freshly prepared human red blood cells obtained from a healthy donor (100 μL) were incubated at 37°C with a 0.33 mM final lipid concentration, whether complexed or not with plasmid DNA, maintaining for all conditions the 16:1 (mol of lipids: mol of base pairs) ratio. After 4 or 24 h of incubation, samples were centrifuged at 1500X g for 10 min and supernatant absorbance was measured at 414 nm with a Nanodrop ND-100 spectrophotometer. Hemolysis was expressed as a percentage of the hemoglobin release induced by SDS (2% v/v) (positive control, 100% hemolysis). Control experiments were performed measuring the supernatant absorbance of erythrocytes incubated with PBS instead of the cationic lipopolymer [55, 56].

### Viability evaluation in the L-929 and Vero cell lines

To analyze possible cytotoxic effects, cell viability was measured as the mean of the activity of mitochondrial succinate dehydrogenase, using the tetrazolium salt MTT [57]. The L-929 and Vero cell lines were obtained from Asociación Banco Argentino de Células (ABAC), Argentina. L-929 and Vero cells were seeded in a 96-well plate at 2 x 10^4^ cells/well density. Cells were cultured with 150 μL MEM/EBSS NEAA (HyClone) modified medium prepared according to the manufacturer’s instructions and supplemented with penicillin (100 U/mL), streptomycin (100 μg/mL), amphotericin B (0.25 μg/mL) and 10% v/v FBS. The plate was incubated in a 5% CO_2_ atmosphere for 24 h at 37°C. Afterwards, at 90% cell confluence, the medium was removed and cells were cultured in the presence of different concentrations (0.5, 1 and 1.5 mM) of polymerized DMPC:DC_8,9_PC:DOTAP (1:1:0.2), DMPC:DC_8,9_PC:SA (1:1:0.2) or DMPC:DC_8,9_PC:MCL (1:1:0.2) with or without pDsRed plasmid DNA diluted in maintenance medium (same medium as described above but supplemented with 1% FBS instead of 10%). For all the conditions assayed, the 16:1 (mol of lipids: mol of base pairs) ratio was maintained. After a 23-h incubation, the culture medium was removed and cells were washed twice with PBS and incubated for 2 h in a 5% CO_2_ atmosphere at 37°C with a 0.5 mg/mL MTT solution prepared in maintenance medium [57]. Supernatants were discarded and cells were homogenized with 200 μL ethanol 95% v/v. Absorbance at 595 nm was determined using a microplate reader (MRXTC Dynex Technologies). Cells incubated only with maintenance medium were used as a control. The absorbance obtained from this control was taken as 100% cell viability and sample data adjusted to this value.

### Statistical analysis

Results are expressed as mean ± SE. Statistical analysis was performed using Graph Pad Prism v6.0 software. The different statistical tests used are detailed within the presented results. Differences were considered to be significant when p < 0.05.

## Results

### Preparation of cationic lipopolymers

**Fig 1:**
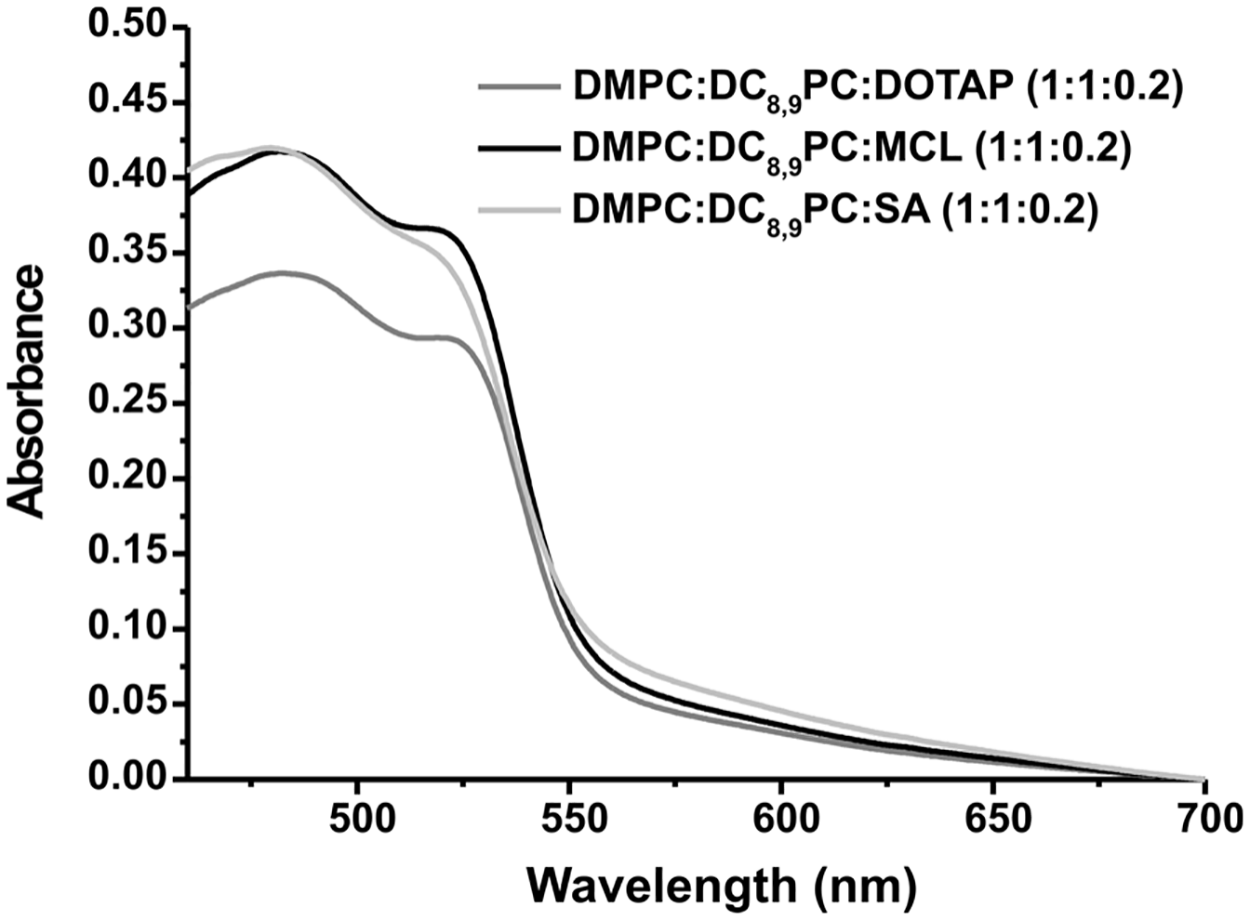
Polymerization confirmation. Absorbance as a function of wavelength (nm) for the DMPC:DC_8,9_PC:DOTAP (1:1:0.2), DMPC:DC_8,9_PC:MCL (1:1:0.2) and DMPC:DC_8,9_PC:SA (1:1:0.2) mixtures, prepared with the copolymerization methodology, after 20 UV irradiation cycles. Peaks observed around 480 and 520 nm are indicative of polymer formation.

The polymerization of the DMPC:DC_8,9_PC 1:1 mixture has been previously described in Alonso-Romanowski *et al*. (2003) and Temprana *et al*. (2010 and 2011) [34, 47, 49]. In these works, the polymerization process was followed by measuring the absorbance in the visible region after 20 polymerization cycles [34, 47, 58, 59]. Fig 1 shows the visible spectra obtained for the DMPC:DC_8,9_PC:DOTAP (1:1:0.2), DMPC:DC_8,9_PC:SA (1:1:0.2), and DMPC:DC_8,9_PC:MCL (1:1:0.2) mixtures, prepared with the copolymerization methodology after 20 irradiation cycles.

As seen in Fig 1, the peaks observed around 480 and 520 nm are indicative of polymer formation [34], whereas the absence of absorbance at λ ~ 610 nm indicates that vesicles and not tubules were present in the suspension [34, 60].

### Size measurements

Since the presence of the CL did not affect DC_8,9_PC polymerization in the copolymerization methodology, the effective diameter and size stability of the cationic lipopolymer were used to choose the best methodology for the preparation of cationic lipopolymers. Table 1 shows the effective diameter obtained for the DMPC:DC_8,9_PC:DOTAP (1:1:0.2), DMPC:DC_8,9_PC:SA (1:1:0.2) and DMPC:DC_8,9_PC:MCL (1:1:0.2) mixtures prepared with the copolymerization and the cationic addition technique, and the size stability after a 166-day incubation at 4°C.

**Table 1:** Light-scattering measurements.

Formulation	Cationic addition		Copolymerization	
	Day 1 (nm)	Day 166 (nm)	Day 1 (nm)	Day 166 (nm)
DMPC:DC_8,9_PC:DOTAP(1:1:0.2)	565 ± 57	519 ± 13	154 ± 7 **	106 ± 25 **
DMPC:DC_8,9_PC:SA(1:1:0.2)	1162 ± 274 Δ	1129 ± 103 Δ	157 ± 6 ****	251 ± 3 ****
DMPC:DC_8,9_PC:MCL(1:1:0.2)	611 ± 49	468 ± 15	168 ± 11 **	182 ± 3

Cationic lipopolymer diameters, measured by light-scattering, after a 1- or 166-day incubation period at 4°C. Values are the means of three determinations ± standard error (SE). Statistical analysis was performed by Two-Way ANOVA with Tukey´s multiple comparisons post-test.

**<0.01;

****<0.0001 (when the same day but different methodology are compared within the same cationic lipopolymer).

Δ<0.001 (when the same day but different cationic lipopolymer are compared within the same methodology). Other values are not statistically different.

The vesicle sizes obtained for all the formulations with the cationic addition technique were higher than those obtained for the same formulation with the copolymerization methodology (Table 1). After 166 days at 4°C, in the case of DMPC:DC_8,9_PC:DOTAP (1:1:0.2), the diameters of the cationic lipopolymers changed from 154 ± 7 nm to 106 ± 25 nm (representing a size decrease of around 31%), whereas in the case of DMPC:DC_8,9_PC:SA (1:1:0.2), they changed from 157 ± 6 nm to 251 ± 3 nm (representing a size increase of around 60%) for the copolymerized samples. No significant size change was observed for the DMPC:DC_8,9_PC:MCL (1:1:0.2) sample prepared with the copolymerization methodology. On the other hand, this sample was the only one that presented a size decrease of approximately 23% when the cationic addition methodology was used (Table 1). In light of these results, the cationic lipopolymers made with the copolymerization methodology were used in further experiments.

### Gel retardation assay

**Fig 2:**
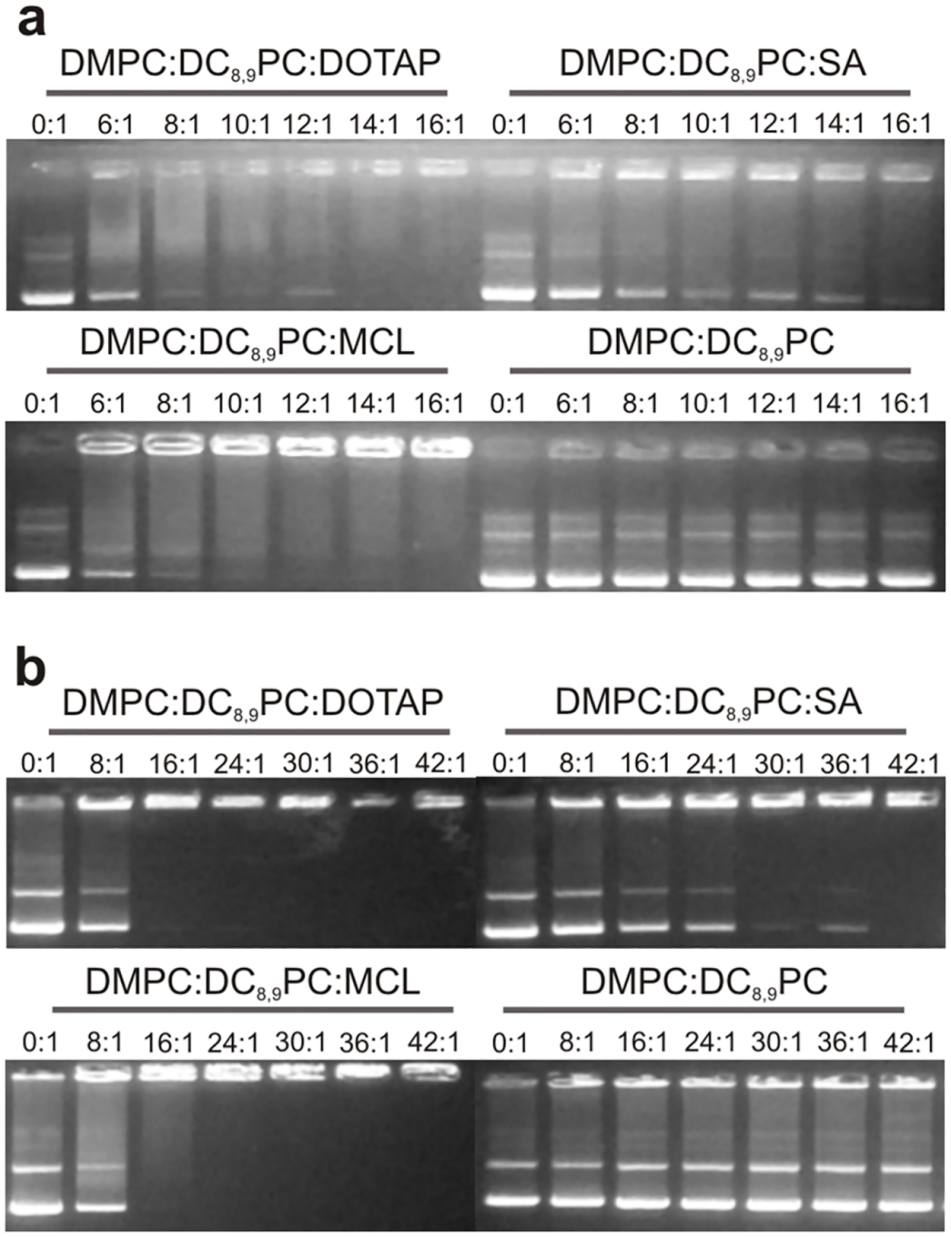
Study of cationic lipopolymer/DNA interaction. Gel retardation assay for (a) DMPC:DC_8,9_PC:DOTAP (1:1:0.2), DMPC:DC_8,9_PC:SA (1:1:0.2), DMPC:DC_8,9_PC:MCL (1:1:0.2) and DMPC:DC_8,9_PC (1:1) mixtures incubated at 0:1, 6:1, 8:1, 10:1, 12:1, 14:1, 16:1 cationic lipopolymer or lipopolymer/pCH110 plasmid DNA ratios (mol of lipids: mol of base pairs) and (b) DMPC:DC_8,9_PC:DOTAP (1:1:0.2), DMPC:DC_8,9_PC:SA (1:1:0.2), DMPC:DC_8,9_PC:MCL (1:1:0.2) and DMPC:DC_8,9_PC (1:1) mixtures incubated at 0:1, 8:1, 16:1, 24:1, 30:1, 36:1, 42:1 cationic lipopolymer or lipopolymer/pDsRed plasmid DNA ratios (mol of lipids: mol of base pairs). All lanes were loaded with 1 μg of plasmid DNA.

To investigate the stoichiometry of the cationic lipopolymer/DNA complex and the effect of plasmid size, a gel retardation assay was performed [54, 61–64]. Different cationic lipopolymer/DNA ratios (mol of lipids: mol of base pairs) were evaluated maintaining constant the amount of DNA (1 μg), both for the pCH110 and pDsRed plasmid DNAs. Fig 2a shows the results obtained for the pCH110 plasmid and Fig 2b shows those obtained for the pDsRed plasmid.

**Fig 3:**
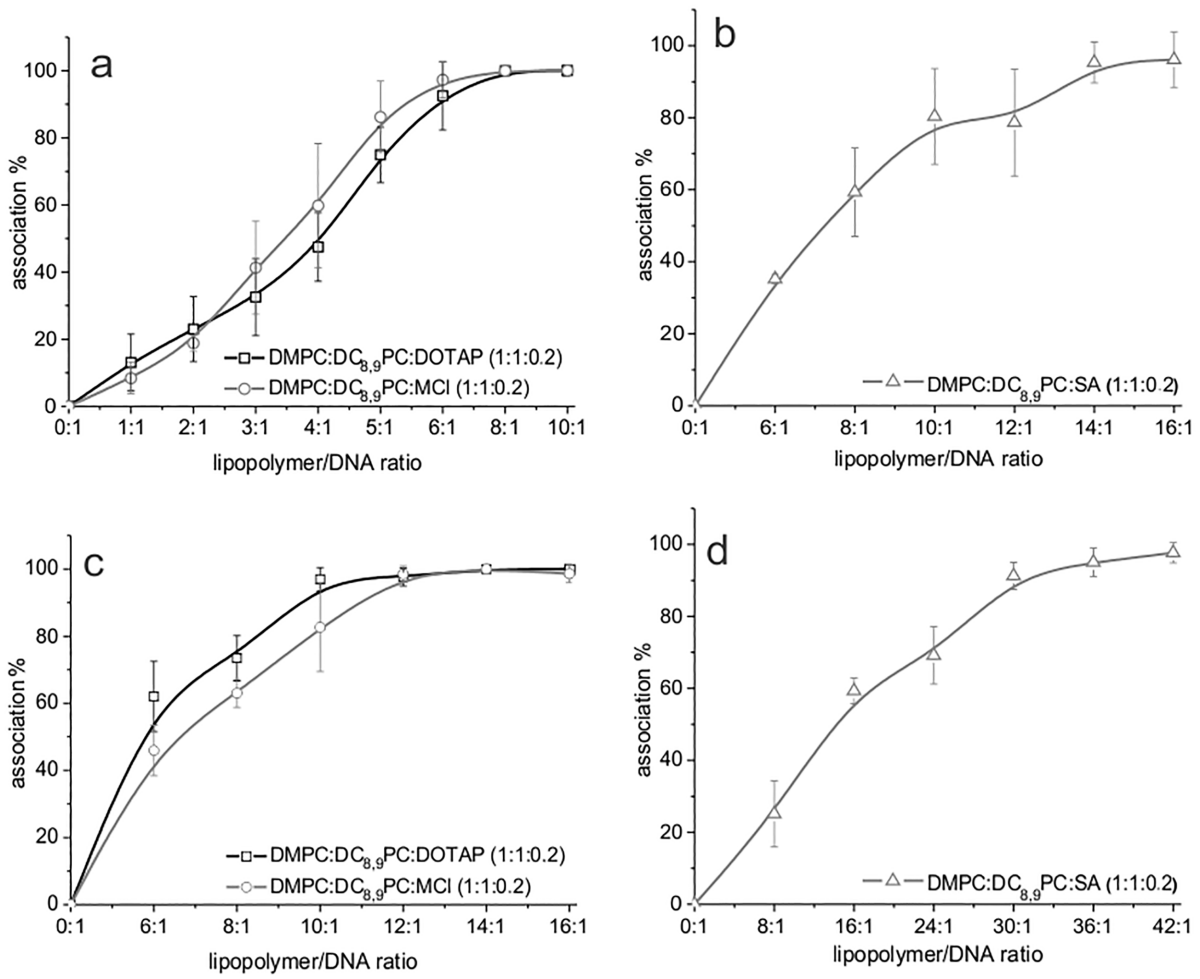
Stoichiometry of the cationic lipopolymer/DNA complex. Percentage of plasmid DNA association as a function of cationic lipopolymer/pCH110 (a and b) or pDsRed (c and d) plasmid DNA ratios (mol of lipids: mol of base pairs) for the DMPC:DC_8,9_PC:DOTAP (1:1:0.2), DMPC:DC_8,9_PC:SA (1:1:0.2), and DMPC:DC_8,9_PC:MCL (1:1:0.2) formulations.

DMPC:DC_8,9_PC (1:1) did not interact with plasmid DNA since, independently of the plasmid (pCH110 or pDsRed) and the lipopolymer/DNA ratio used, the plasmid migrated in the same way as the control without lipids (Fig 2). In this sense, the addition of a positive charge through CL allowed this interaction to occur, although the association efficiency was found to be formulation-dependent. To gain further insight in the stoichiometry of the complex, different cationic lipopolymer/DNA ratios were tested, and the results obtained for both plasmids were analyzed by band densitometry analysis (see Methods, section *Gel Retardation Assay*). The results obtained for the different formulations and plasmids are plotted as association percentage versus cationic lipopolymer/DNA ratio (Fig 3).

It is important to note that the cationic lipopolymer DMPC:DC_8,9_PC:SA (1:1:0.2) was the least efficient in complexing DNA when compared to DMPC:DC_8,9_PC:DOTAP (1:1:0.2) and DMPC:DC_8,9_PC:MCL (1:1:0.2), independently of the size of the plasmid used (see Fig 3a and 3b for pCH110 plasmid and Fig 3c and 3d for pDsRed). In particular, the cationic lipopolymer/pDsRed association was complete for ratios (mol of lipids: mol of base pairs) equal to or greater than 12:1 for DMPC:DC_8.9_PC:DOTAP (1:1:0.2) and DMPC:DC_8.9_PC:MCL (1:1:0.2) and greater than 42:1 for DMPC:DC_8.9_PC:SA (1:1:0.2).

### Z-potential

Since the polar head groups and the hydrophobic moieties presented differences among the CL used and considering that this fact can have effects on the surface charges of the lipopolymers, we next determined the Z-potential of the cationic lipopolymers obtained. Table 2 shows the Z-potential values obtained for the formulated cationic lipopolymers. We found no statistical differences in the surface charge of the different cationic lipopolymer formulations.

**Table 2:** Z-potential measurements.

Formulation	Z-potential (mV)
DMPC:DC_8,9_PC:DOTAP(1:1:0.2)	33.3 ± 0.8
DMPC:DC_8,9_PC:SA(1:1:0.2)	31.8 ± 0.4
DMPC:DC_8,9_PC:MCL(1:1:0.2)	32.4 ± 0.6

Z-potential of cationic lipopolymers. Values are the means of three determinations ± standard error (SE). Statistical analysis was performed by One-Way ANOVA with Tukey´s multiple comparisons post-test. Values are not statistically different.

### Effect of different incubation media on the cationic lipopolymer/DNA interaction

**Fig 4:**
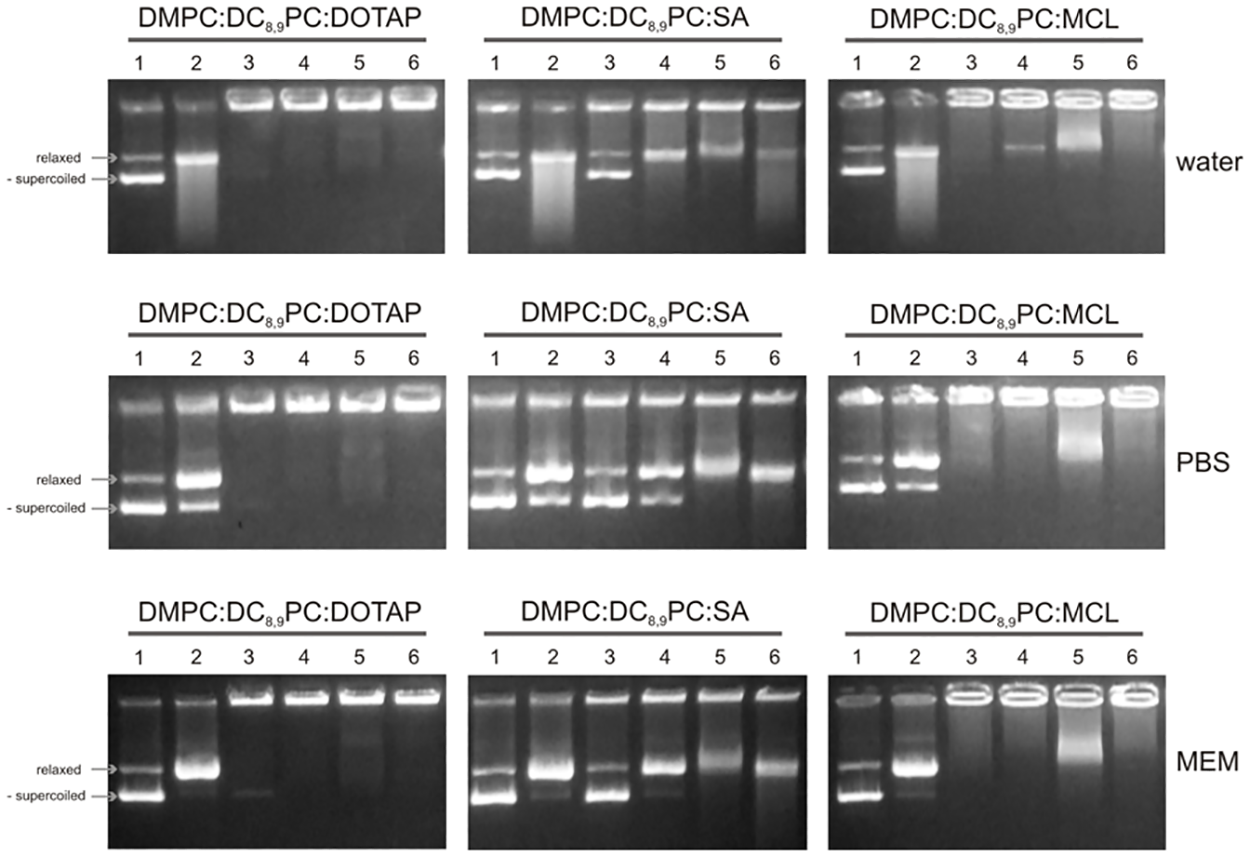
Effect of different incubation media on the cationic lipopolymer/DNA interaction. The different cationic lipopolymers were incubated with the pDsRed plasmid DNA in a 16:1 (mol of lipids: mol of base pairs) ratio in the medium (water, PBS or MEM) indicated on the right. Each lane was loaded with 1 μg of plasmid DNA. Lanes correspond to: (1) pDsRed alone with an additional 10-min incubation in the indicated medium, (2) pDsRed alone with an additional 10-min incubation in the presence of 10% v/v FBS, (3) cationic lipopolymer (stated above the gel picture)/pDsRed plasmid DNA complex formed in the indicated medium with an additional 10-min incubation without FBS, (4) cationic lipopolymer (stated above the gel picture)/pDsRed plasmid DNA complex formed in the indicated medium with an additional 10-min incubation in the presence of 10% v/v FBS, (5) cationic lipopolymer (stated above the gel picture)/pDsRed plasmid DNA complex formed in the indicated medium with an additional 10-min incubation in the presence of 50% v/v FBS, (6) cationic lipopolymer (stated above the gel picture)/pDsRed plasmid DNA complex formed in the indicated medium with 10% v/v FBS and with an additional 10-min incubation in the presence of 50% v/v FBS. The two main topological plasmid conformations, relaxed and negatively supercoiled, are indicated with arrows on the left of the figure noted as relax and -supercoiled, respectively.

To study the effect of the incubation media on the formation of cationic lipopolymer/DNA complexes, the complexes (16:1 mol of lipids: mol of base pairs) were formed in water, PBS or cell culture medium MEM, with or without FBS (10% v/v final concentration) and then incubated for additional 10 min either in the presence or in the absence of 10% or 50% v/v FBS. The results obtained are shown in Fig 4.

It is important to mention that, for comparison, the ratio used for all the formulations was 16:1 (mol of lipids: mol of base pairs ratio), although the formulation containing the CL SA, at this ratio, could not complex all the pDsRed plasmid DNA (Fig 3d) (see “free” and/or degraded plasmid DNA present in all lanes of the gels corresponding to the DMPC:DC_8,9_PC:SA (1:1:0.2) formulation in Fig 4). Note that small amounts of serum DNAses in a short-time incubation (10 min) turned the negatively supercoiled plasmid conformation into the relaxed form (see for example Fig 4, DMPC:DC_8,9_PC:DOTAP gel, PBS medium, lanes 1 and 2). Higher serum DNAses activity resulted into a diffuse smear, seen in the gel lane (see for example Fig 4, DMPC:DC_8,9_PC:DOTAP gel, water medium, lane 2). As seen in Fig 4, the DMPC:DC_8,9_PC:DOTAP (1:1:0.2) formulation efficiently complexed the pDsRed plasmid in all the media tested, and almost no degradation occurred after the 10-min 50% v/v serum incubation (see lane 6, gels corresponding to the DMPC:DC_8,9_PC:DOTAP (1:1:0.2) formulation). This was not the case for the DMPC:DC_8,9_PC:MCL (1:1:0.2) formulation, in which little DNA degradation occurred after the 10-min 50% v/v serum incubation in all the media tested (compare Fig 4, lane 6 of the gels corresponding to the DMPC:DC_8,9_PC:DOTAP (1:1:0.2) and DMPC:DC_8,9_PC:MCL (1:1:0.2) formulations).

### Serum nucleases digestion assay

**Fig 5:**
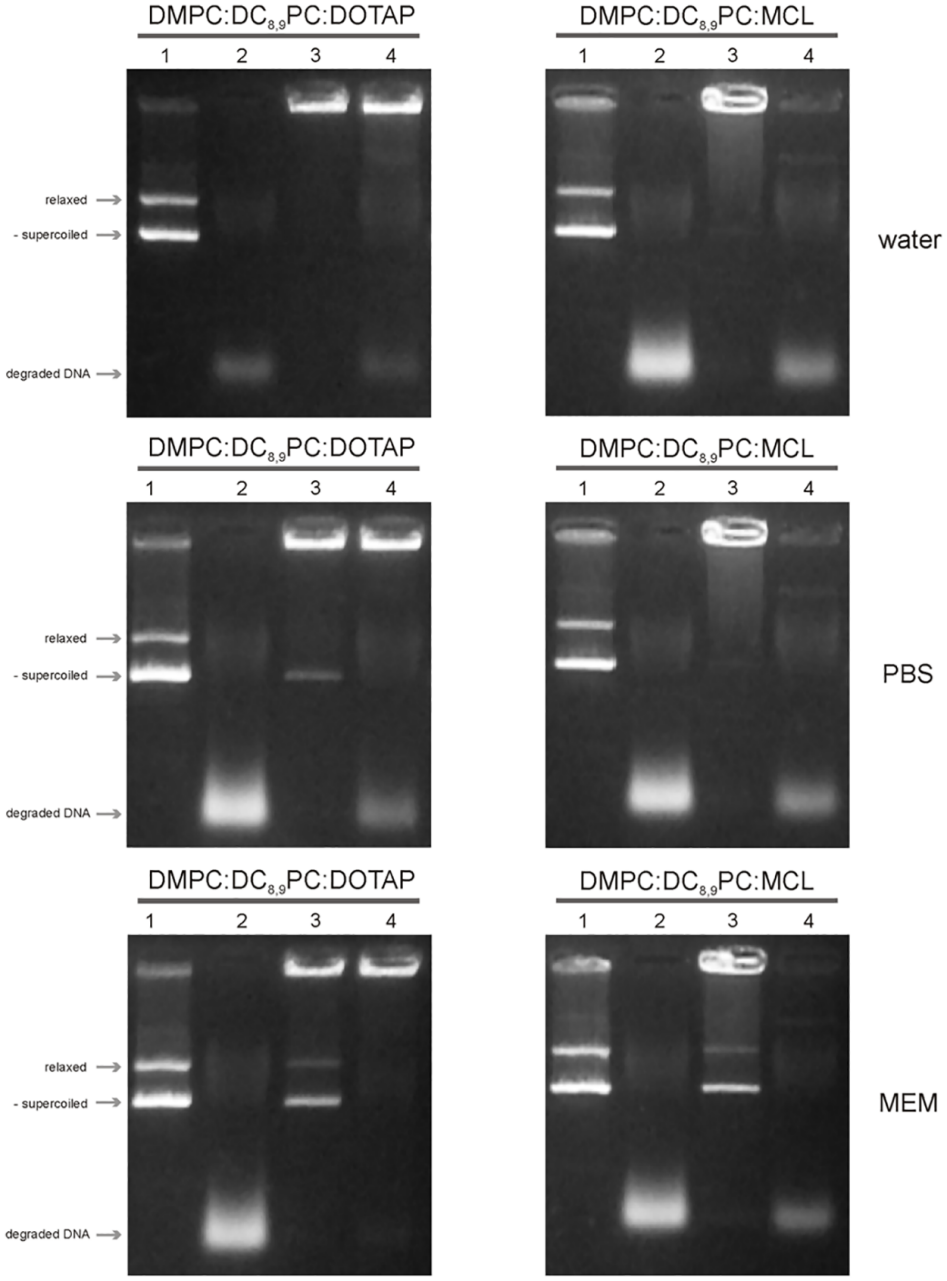
Serum nucleases digestion assay. The different cationic lipopolymers DMPC:DC_8,9_PC:DOTAP (1:1:0.2) and DMPC:DC_8,9_PC:MCL (1:1:0.2)/pDsRed plasmid DNA complexes (16:1 mol of lipids: mol of base pairs ratio) were formed in water, PBS or MEM (indicated on the right). Each lane was loaded with 1 μg of plasmid DNA. Lanes correspond to: (1) pDsRed alone with a 24-h incubation in the indicated medium, (2) pDsRed alone with a 24-h incubation in the presence of 50% v/v FBS, (3) cationic lipopolymer (stated above the gel picture)/pDsRed plasmid DNA complex formed in the indicated medium with a 24-h incubation in the indicated medium without FBS, and (4) cationic lipopolymer (stated above the gel picture)/pDsRed plasmid DNA complex formed in the indicated medium with a 24-h incubation in the presence of 50% v/v FBS. The two main topological plasmid conformations, relaxed and negatively supercoiled, are indicated with arrows on the left of the figure noted as relax and -supercoiled, respectively. Degraded DNA is also indicated with an arrow on the left of the figure.

The results obtained regarding the effect of different incubation media on the cationic lipopolymer/DNA interaction suggested a good performance in protecting DNA from degradation by the DMPC:DC_8,9_PC:DOTAP (1:1:0.2) formulation followed, although less efficiently, by the DMPC:DC_8,9_PC:MCL (1:1:0.2) formulation. However, a 10-min incubation might not be enough to test serum stability and protection capacity. Thus, to evaluate the cationic lipopolymer/DNA stability and cationic lipopolymer protection capacity of the DMPC:DC_8,9_PC:DOTAP (1:1:0.2) and DMPC:DC_8,9_PC:MCL (1:1:0.2) formulations, a 24-h incubation at 37°C in a 50% v/v FBS solution was carried out for complexes formed in water, PBS and MEM. Results obtained for the DMPC:DC_8,9_PC:DOTAP (1:1:0.2) and DMPC:DC_8,9_PC:MCL (1:1:0.2) formulations are shown in Fig 5.

When the pDsRed plasmid was not complexed with cationic lipopolymers, DNA was totally degraded after the 24-h incubation in the presence of 50% v/v FBS in all the media tested (see Fig 5, lane 2, and compare with non-degraded plasmid DNA control in lane 1). “Free” plasmid was found in both formulations (DMPC:DC_8,9_PC:DOTAP (1:1:0.2) and DMPC:DC_8,9_PC:MCL (1:1:0.2)) after the 24-h incubation without serum in PBS and MEM, although a higher amount of “free” plasmid was found in the latter (Fig 5, lane 3). Finally, total DNA degradation was observed in the case of the DMPC:DC_8,9_PC:MCL (1:1:0.2) formulation, with almost no detectable complexes present at the loading well (see Fig 5, lane 4, gels corresponding to the DMPC:DC_8,9_PC:MCL (1:1:0.2) formulation). This was not the case for the DNA complexed with the DMPC:DC_8,9_PC:DOTAP (1:1:0.2) formulation, in which little DNA degradation occurred (see Fig 5, lane 4, gels corresponding to the DMPC:DC_8,9_PC:DOTAP (1:1:0.2) formulation).

### Flow cytometry

Flow cytometry can be used for the characterization of liposomal suspensions if the liposomes are labeled with some type of fluorescent probe within the membrane [65–67]. In our work, we found this approach very interesting since the developed cationic lipopolymers are fluorescent *per se* (61) and DNA can be labeled with SYBR Green. Thus, double fluorescence is indicative of the presence of both the cationic lipopolymer and DNA.

**Fig 6:**
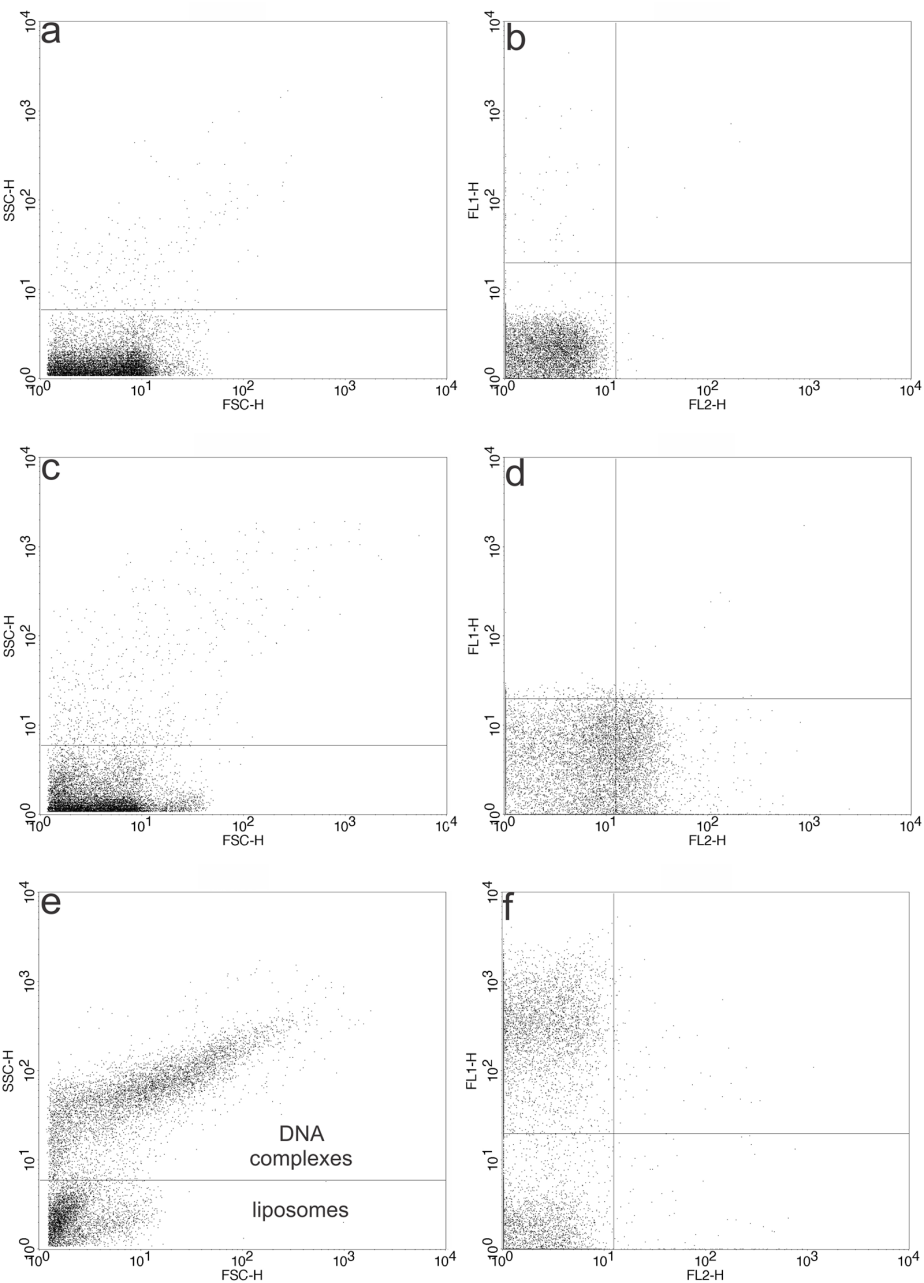
Optimization of the study of cationic lipopolymer/DNA interaction by flow cytometry. Flow cytometry analysis results for non-polymerized DMPC:DC_8,9_PC:MCL (1:1:0.2) liposomes, used to set control values of (a) SSC-H values (related to particle complexity) versus FSC-H values (related to particle size) and (b) FL1 values (where SYBR^®^ Green I-labeled plasmid DNA fluorescence is detected) and FL2 values (where cationic lipopolymer fluorescence is detected). (c) and (d) show the results for polymerized DMPC:DC_8,9_PC:MCL (1:1:0.2) for SSC-H versus FSC-H and FL2 versus FL1 values respectively. (e) and (f) show the results for non-polymerized DMPC:DC_8,9_PC:MCL (1:1:0.2)/SYBR^®^ Green I-labeled pDsRed plasmid DNA complexes for SSC-H and FSC-H values and FL2 versus FL1 values respectively.

So, we study the formation of complexes, with the advantage that the cationic lipopolymer/DNA interaction is not altered by any probe within the membrane, usually used to study this kind of complexes [68]. Non-polymerized vesicles were used to set control values of SSC-H values (related to particle complexity), FSC-H values (related to particle size), FL1 (where SYBR^®^ Green I-labeled plasmid DNA fluorescence is detected) and FL2 (where cationic lipopolymer fluorescence is detected). As an example, results obtained for non-polymerized DMPC:DC_8,9_PC:MCL (1:1:0.2) liposomes for FSC-H versus SSC-H and FL2 versus FL1 are shown in Fig 6a and 6b, respectively. Fig 6c shows that the SSC-H and FSC-H values found for polymerized DMPC:DC_8,9_PC:MCL (1:1:0.2) samples were very similar to those obtained for non-polymerized ones (Fig 6a). On the other hand, after polymerization, FL2 fluorescence values were increased (compare Fig 6d with Fig 6b). Since fluorescence in the FL1 channel was also increased, this last value was used to set the negative fluorescence for this channel. SYBR^®^ Green I-labeled DNA was incubated with non-polymerized DMPC:DC_8,9_PC:MCL (1:1:0.2) liposomes to set positive fluorescence in the FL1 channel. The results obtained for FSC-H versus SSC-H and FL2 versus FL1 are shown in Fig 6e and 6f, respectively. In this case, two populations were found when FSC-H and SSC-H were analyzed: one corresponding to liposomes alone, presenting values similar to those found for the control (Fig 6a), and the other one with higher FSC-H and SSC-H values, suggesting liposome/DNA complex formation presenting heterogeneity in complexity and sizes (Fig 6e). As seen in Fig 6f, two populations were detected: one presenting high fluorescence values in the FL1 channel (corresponding to complexes containing SYBR^®^ Green I-labeled DNA), and the other non-fluorescent one corresponding to liposomes alone.

**Fig 7:**
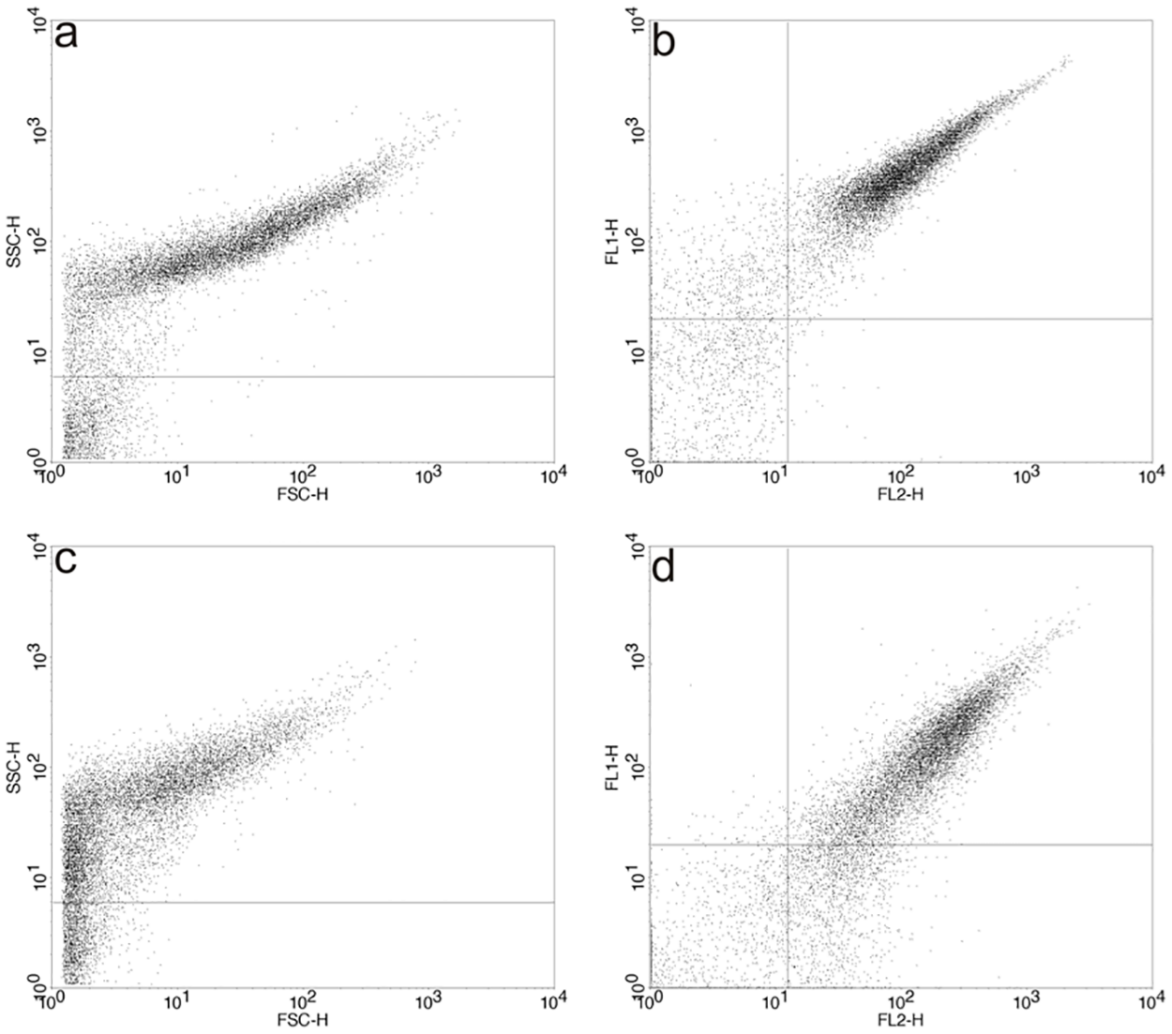
Study of cationic lipopolymer/DNA interaction by flow cytometry. Flow cytometry analysis results for (a) SSC-H versus FSC-H and (b) FL2 versus FL1 values for polymerized DMPC:DC_8,9_PC:DOTAP (1:1:0.2)/SYBR^®^ Green I-labeled pDsRed plasmid DNA complexes and (c) SSC-H versus FSC-H and (d) FL2 versus FL1 values for polymerized DMPC:DC_8,9_PC:MCL (1:1:0.2)/SYBR^®^ Green I-labeled pDsRed plasmid DNA complexes.

The results obtained for FSC-H versus SSC-H and FL2 versus FL1 for the complexes formed with DMPC:DC_8,9_PC:DOTAP (1:1:0.2) and SYBR^®^ Green I-labeled DNA are shown in Fig 7a and 7b respectively, whereas those obtained for FSC-H versus SSC-H and FL2 versus FL1 for DMPC:DC_8,9_PC:MCL (1:1:0.2) complexed with SYBR^®^ Green I-labeled DNA are shown in Fig 7c and 7d, respectively. Both formulations presented a similar complexity (SSC-H values) and size (FSC-H values) distribution (see Fig 7a for complexes formed with DMPC:DC_8,9_PC:DOTAP (1:1:0.2) lipopolymer and Fig 7c for complexes formed with DMPC:DC_8,9_PC:MCL (1:1:0.2) lipopolymer). It is important to remark that events with double positive fluorescence were found for complexes formed with both DMPC:DC_8,9_PC:DOTAP (1:1:0.2) and DMPC:DC_8,9_PC:MCL (1:1:0.2) lipopolymers (Fig 7b and 7d, respectively), meaning that the SYBR^®^ Green I-labeled plasmid DNA is complexed with the two cationic lipopolymers used.

**Fig 8:**
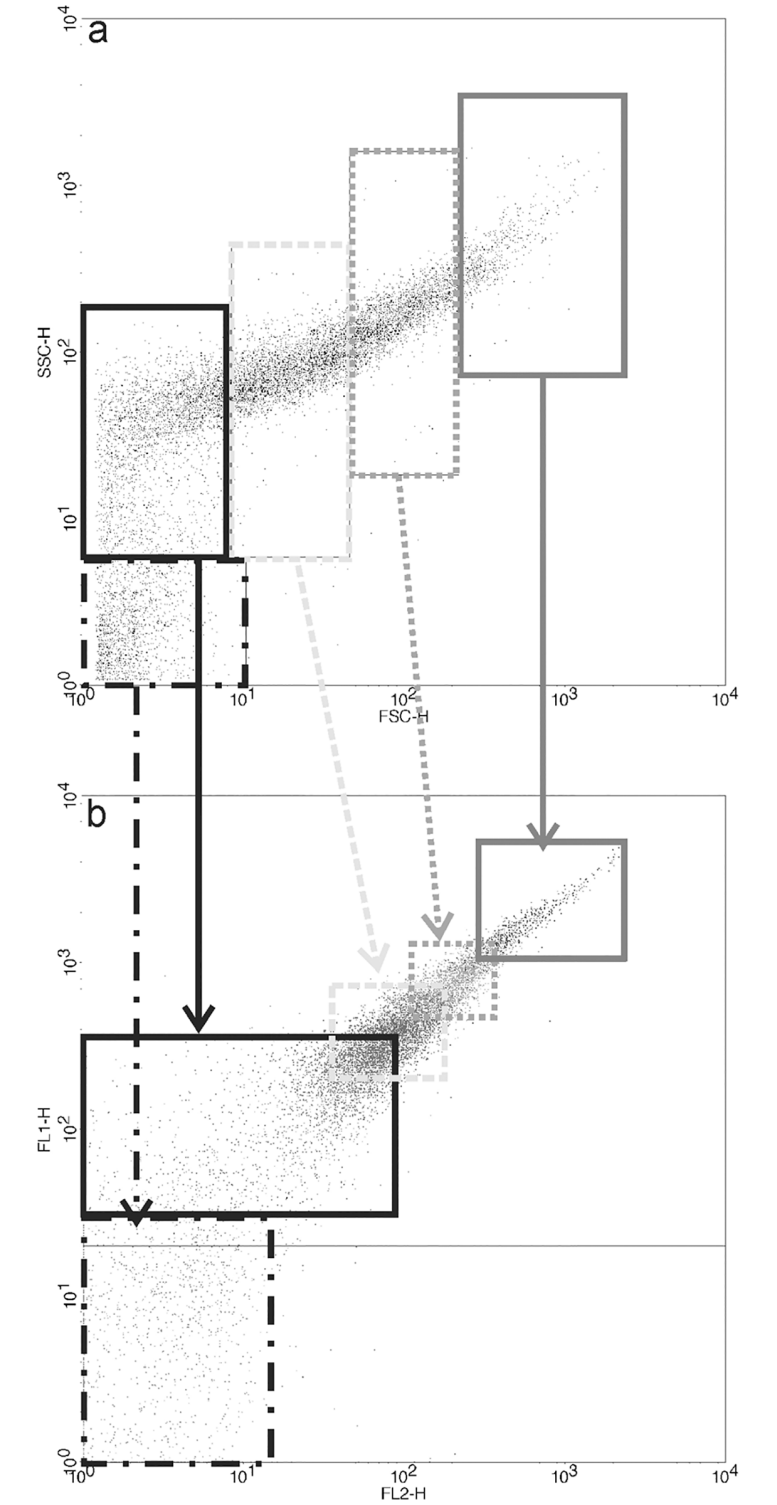
Study of cationic lipopolymer/DNA interaction by flow cytometry. Flow cytometry analysis results for (a) SSC-H versus FSC-H and (b) FL2 versus FL1 values for polymerized DMPC:DC_8,9_PC:DOTAP (1:1:0.2)/SYBR^®^ Green I-labeled pDsRed plasmid DNA complexes. Different regions were drawn based on SSC-H and FSC-H values (a) and the fluorescence values for FL1 and FL2 corresponding to each region are marked in the FL2 versus FL1 graph (b) with the same borderline shown by the same style line arrow.

To analyze this result in depth, different regions were drawn based on the SSC-H and FSC-H values, and the FL1 and FL2 fluorescence of the events within these regions were analyzed. Fig 8 shows the results obtained for complexes formed with DMPC:DC_8,9_PC:DOTAP (1:1:0.2) and SYBR^®^ Green I-labeled pDsRed plasmid. Note that the higher the complexity and size values, the higher the fluorescence in the FL1 and FL2 channels (Fig 8a and 8b). Similar results were found for DMPC:DC_8,9_PC:MCL (1:1:0.2) and the pDsRed plasmid (data not shown).

### Hemolysis and viability evaluation in the L-929 and Vero cell lines

The first approach to evaluate the possible cytotoxic effect of the different formulations, either complexed or not with plasmid DNA, was through the determination of the ability to induce human red blood cell hemolysis. Hemolysis results showed no significant differences between the three different cationic lipopolymers, either complexed or not with DNA, when compared to a control experiment after a 4- and 24-h incubation (Table 3).

**Table 3:** Hemolysis percentage (% H).

Formulation	% H(4 hours)	% H(24 hours)
PBS	0.41 ± 0.05	2.43 ± 1.18
DMPC:DC_8,9_PC:DOTAP(1:1:0.2)	0.30 ± 0.08	2.53 ± 1.36
DMPC:DC_8,9_PC:SA(1:1:0.2)	0.43 ± 0.13	3.67 ± 0.83 **
DMPC:DC_8,9_PC:MCL(1:1:0.2)	0.33 ± 0.05	5.80 ± 2.29 ****
DMPC:DC_8,9_PC:DOTAP(1:1:0.2)/pDsRed (16:1)	0.33 ± 0.10	3.99 ± 0.88 **
DMPC:DC_8,9_PC:SA(1:1:0.2)/pDsRed (16:1)	0.41 ± 0.17	3.19 ± 1.65 *
DMPC:DC_8,9_PC:MCL(1:1:0.2)/pDsRed (16:1)	0.45 ± 0.08	4.16 ± 0.70 **

Hemolysis results after incubation periods of 4 or 24 h. Values are the means of five determinations ± standard error (SE). Statistical analysis was performed by Two-Way ANOVA with Sidak´s multiple comparisons post-test.

*<0.05;

**<0.01;

****<0.0001 (when different incubation times are compared within the same formulation). Other values are not statistically different.

**Fig 9:**
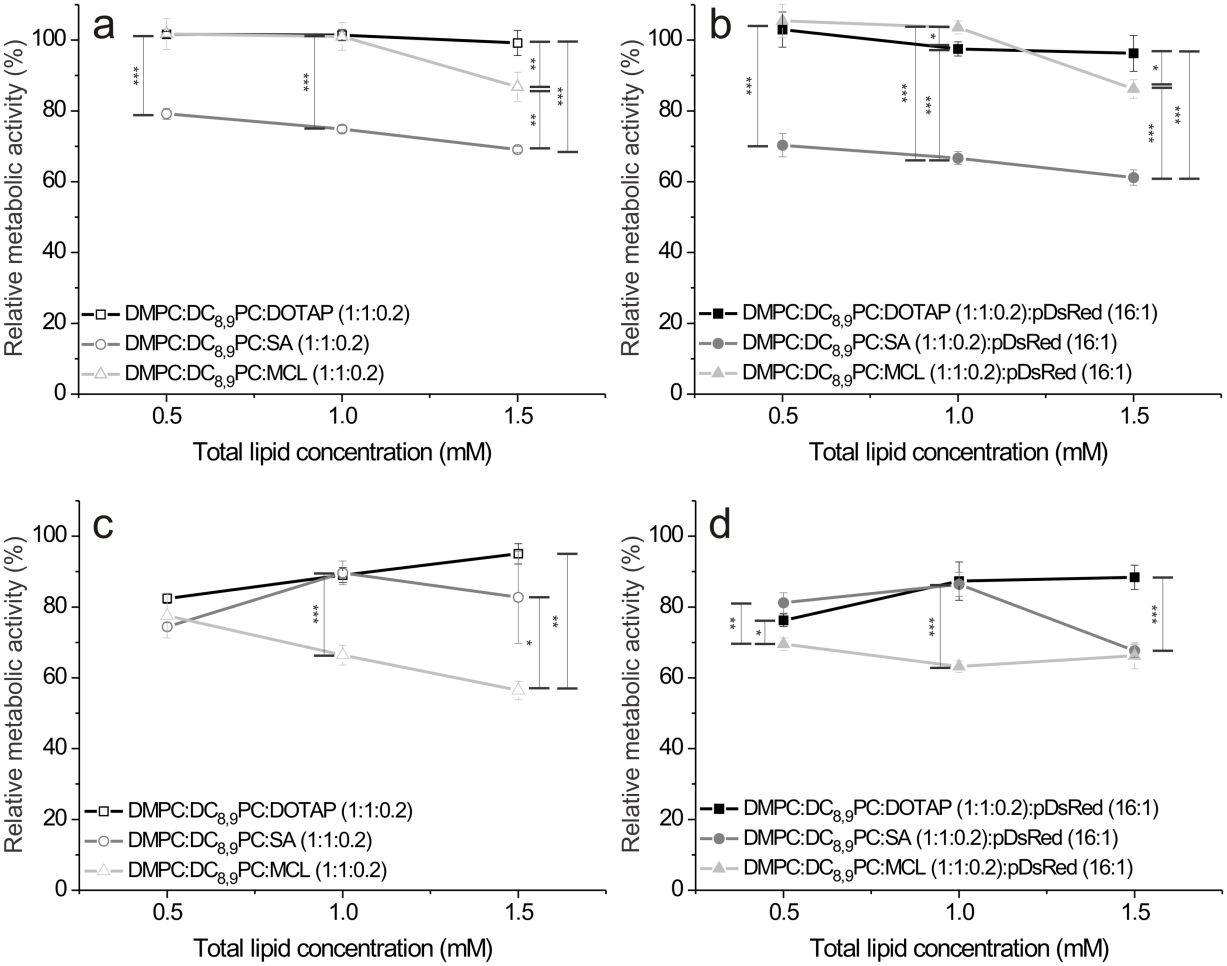
Cytotoxicity determination. Cell viability percentage as a function of total lipid concentration (mM) for L929 cells incubated with cationic lipopolymers alone (a) or complexed with pDsRed plasmid DNA in a 16:1 (mol of lipids: mol of base pairs) ratio (b); and for Vero cells incubated with cationic lipopolymers alone (c) or complexed with pDsRed plasmid DNA in a 16:1 (mol of lipids: mol of base pairs) ratio (d). Statistical analysis was performed by Two-Way ANOVA with Tukey´s multiple comparisons post-test. *<0.05; **<0.01; ***<0.001; ****<0.0001.

The L929 and Vero cell lines were used as models to test the toxicity of cationic lipopolymers, either complexed or not with plasmid DNA. The results obtained with the L929 cell line after incubation with the DMPC:DC_8,9_PC:DOTAP (1:1:0.2), DMPC:DC_8,9_PC:SA (1:1:0.2) and DMPC:DC_8,9_PC:MCL (1:1:0.2) lipopolymers either complexed or not with the pDsRed plasmid DNA are shown in Fig 9a and 9b, respectively, whereas those obtained with the Vero cell line after treatment with the DMPC:DC_8,9_PC:DOTAP (1:1:0.2), DMPC:DC_8,9_PC:SA (1:1:0.2) and DMPC:DC_8,9_PC:MCL (1:1:0.2) lipopolymers are shown in Fig 9c (without DNA) and 9 d (with DNA). As seen in Fig 9a and 9b, no important differences in cell viability were observed between cationic lipopolymers alone and cationic lipopolymer/DNA complexes for the DMPC:DC_8,9_PC:DOTAP (1:1:0.2) and DMPC:DC_8,9_PC:MCL (1:1:0.2) formulations in the L929 cell line; and around a 10% cell viability reduction was only observed for the DMPC:DC_8,9_PC:MCL (1:1:0.2) formulation at the highest concentration assayed. This was not the case for DMPC:DC_8,9_PC:SA (1:1:0.2) lipopolymers which affected L929 cell viability in approximately 20 to 30% without DNA and in approximately 30 to 40% when complexed with plasmid DNA (Fig 9a and 9b). In the case of the Vero cell line, the three cationic lipopolymers presented a cell viability reduction of approximately 20% at the lowest concentration assayed. Increasing the total lipid concentration did not result in a higher cytotoxic effect for the DMPC:DC_8,9_PC:DOTAP (1:1:0.2) and DMPC:DC_8,9_PC:SA (1:1:0.2) formulations (Fig 9c). This was not the case for the DMPC:DC_8,9_PC:MCL (1:1:0.2) formulation, which presented a cell viability reduction of approximately 40% at 1.5 mM total lipid concentration (Fig 9c). Plasmid DNA addition slightly increased the cytotoxic effect for the DMPC:DC_8,9_PC:DOTAP (1:1:0.2) and DMPC:DC_8,9_PC:SA (1:1:0.2) formulations and slightly reduced it for the DMPC:DC_8,9_PC:MCL (1:1:0.2) formulation, at the highest total lipid concentration used (Fig 9d).

## Discussion

### Liposome preparation and size measurements

For the DMPC:DC_8,9_PC (1:1) mixture, it has been previously described that, after UV irradiation, conjugated polymers containing alternatively triple, single and double bonds are formed [34, 69]. The polymer backbone presents absorbance in the visible region of the spectrum, and the degree of membrane polymerization is related to both the magnitude and the wavelength of visible absorbance peaks and depends on the number of polymer units electronically coupled [34, 58, 59]. It has also been described that different parameters, including the polymerization process used, the lipid nature and the lipid ratios, may alter the polymerization [49, 70, 71]. Thus, the addition of different lipids to the DMPC:DC_8,9_PC (1:1) mixture might affect the DC_8,9_PC polymerization by disturbing the cross-linking of the adjacent diacetylenic groups [72]. The mixtures containing one of the three different CL (DMPC:DC_8,9_PC:DOTAP (1:1:0.2), DMPC:DC_8,9_PC:SA (1:1:0.2) and DMPC:DC_8,9_PC:MCL (1:1:0.2)) were efficiently polymerized when the CL were included in the formulation (copolymerization process) as determined by visible spectroscopy. Moreover, previously, we published a detailed structural characterization of these formulations containing CL, in which the polymerization efficiency was determined by differential scanning calorimetry [48].

Since the polymerization was not affected by the presence of CL within the formulation, we evaluated the size and the size stability of the cationic lipopolymers obtained with both the copolymerization and the cationic addition techniques, to determine the best methodology to prepare the cationic lipopolymers. As expected, the cationic addition technique led to larger sized cationic lipopolymers when compared to those obtained by the copolymerization technique. In the latter, liposomes were extruded through a 0.2 μm pore polycarbonate membrane and, after that, polymerized. Thus, the diameter sizes of the unilamellar vesicles obtained were found to be less than 200 nm. On the other hand, in the addition technique, where extruded lipopolymers were lyophilized, solubilized in chloroform with the CL, and rehydrated in water after the solvent evaporation, multilamellar liposomes showed larger diameter sizes than those obtained by the copolymerization technique.

It is interesting to point out that, independently of the CL used and the technique used (copolymerization or cationic addition), no drastic changes occurred in the diameter sizes of the cationic lipopolymers after a 166-day incubation period at 4°C. As discussed previously in Temprana *et al*. (2011) [47], polymerization of DMPC:DC_8,9_PC (1:1) lead to a higher system resistance to fusion or aggregation at a 4°C storage condition for 30 days (the effective diameter changed approximately 47%) when compared to the same non-polymerized formulation. In line with this idea, we suggest that CL addition helps to improve this resistance to fusion or aggregation at a 4°C storage condition, although no data are available for the DMPC:DC_8,9_PC (1:1) formulation in this long time period (166 days), and that this improvement might be dependent on the CL used. In this sense, the DMPC:DC_8,9_PC:MCL (1:1:0.2) prepared with the copolymerization technique presented the highest size stability after a 166-day incubation period at 4°C (it changed from 168 ± 11 to 182 ± 3, which represents a change of approximately 8%) among those prepared with the copolymerization technique. This observation is in agreement with Roland *et al*. (2003), who described that a Z potential module greater than 25 mV is correlated with a high stability of the system [73]. In our case, the three formulations presented Z potential values higher than 25 mV.

### Gel retardation assay and Z-potential

To study the interaction between plasmid DNA and the different cationic lipopolymers, a gel retardation assay was performed. This technique allowed demonstrating that DMPC:DC_8,9_PC (1:1) does not interact with plasmid DNA. Little is known about the interaction of this kind of lipopolymers and DNA. Chiaramoni *et al*. (2007) [23] described a low association percentage of 11% (of the total amount of plasmid DNA) for the DC_8,9_PC:1,2-dimyristoyl-sn-glycero-3-phosphoethanolamine (DMPE): cholesterol (2:2:1 molar ratio) mixture, although this percentage was determined through a technique different from that used in this work. Likewise, the low interaction could be because lipopolymers without cationic lipids present a Z-potential near to neutrality. Particularly, the DMPC:DC_8,9_PC (1:1) formulation presents a Z-potential value of –8.73 ± 1.01 mV [74]. In this sense, we showed that addition of a CL to the DMPC:DC_8,9_PC (1:1) mixture increased the plasmid DNA association efficiency to a 100%, although the ratio needed to complex the same amount of plasmid DNA varied among the CL used. This interaction between DMPC:DC_8,9_PC:CL (1:1:0.2) formulations and DNA was somehow expected since the DNA/liposome interaction is governed mainly by electrostatic forces [6, 7, 26, 29] and our cationic lipopolymers presented Z-potential values near 30 mV. However, we found that the formulation containing SA was less efficient in plasmid DNA complexing than those containing DOTAP or MCL as cationic lipid, although the three of them presented no significant differences between their Z-potential values. In a previous work, we found distinctive characteristics of the membrane structure of the DMPC:DC_8,9_PC:SA (1:1:0.2) mixture, when compared to those obtained for DMPC:DC_8,9_PC:DOTAP (1:1:0.2) and DMPC:DC_8,9_PC:MCL (1:1:0.2) [48]. The correlation between the membrane structure of these cationic lipopolymers and the plasmid association efficiency is beyond the present discussion, but represents an important issue to be studied and discussed, since it should give important information at the moment of designing new formulations involving the polymerizable lipid DC_8,9_PC.

### Effect of different incubation media on the cationic lipopolymer/DNA interaction and serum nucleases digestion assay

DMPC:DC_8,9_PC:DOTAP (1:1:0.2) and DMPC:DC_8,9_PC:MCL (1:1:0.2) were the most efficient in complexing the plasmid DNA, requiring lesser amounts of lipid to obtain higher DNA association. As serum-containing media might induce DNA degradation by serum nucleases, affecting plasmid integrity, which is a key issue when evaluating possible systems for gene delivery, we studied the complexing and protecting performance of these formulations in different media, with and without serum. The DMPC:DC_8,9_PC:DOTAP (1:1:0.2) formulation was able to efficiently complex the pDsRed plasmid in all the media tested, and almost no degradation occurred after the 10-min 50% v/v serum incubation. Interestingly, this protective effect was also observed after 24-h incubation, with little DNA degradation. This was not the case for the DMPC:DC_8,9_PC:MCL (1:1:0.2) formulation, in which little DNA degradation occurred after the 10-min 50% v/v serum incubation in all the media tested, but total DNA degradation was observed after 24 h, with almost no detectable complexes in all the media tested.

All previously mentioned results suggest that the DMPC:DC_8.9_PC:SA (1:1:0.2) formulation was the least stable and the one with less ability to complex DNA. The DMPC:DC_8.9_PC:MCL (1:1:0.2) formulation showed a better performance, although it was less stable and less effective to protect plasmid DNA than DMPC:DC_8.9_PC:DOTAP (1:1:0.2). This latter formulation was able to protect almost all (depending on the incubation medium) of the plasmid DNA, even against an incubation with 50% v/v FBS for a long time period (24 h at 37°C), proving to be a system with good resistance to serum DNAses. These results are in agreement with those of Moret *et al*. (2001) [75], in which the DOTAP-DNA complex was stable in the presence of DNAse I and in the presence of mouse, rat or human serum.

### Flow cytometry

An interesting approach was to study the cationic lipopolymer/DNA interaction by flow cytometry. This approach has the advantage that the interaction is not altered by any probe within the membrane, due to the intrinsic fluorescence of the lipopolymers. Based on the less efficiency observed for the DMPC:DC_8,9_PC:SA (1:1:0.2) formulation in the interaction with DNA, only DMPC:DC_8.9_PC:DOTAP (1:1:0.2) and DMPC:DC_8,9_PC:MCL (1:1:0.2) were analyzed by flow cytometry. As expected, the cationic lipopolymers alone had a poorly complex population, a relatively conserved size and fluorescence recorded in FL2. When both cationic lipopolymers were incubated with the plasmid DNA pDsRed (either labeled or not), a population with greater complexity and sizes was observed. This allowed confirming that cationic lipopolymers interact with the plasmid pDsRed. The linear fluorescence tendency observed in cationic lipopolymer and DNA complexes suggests that a certain conserved ratio exists and is independent of the final complex size. These results are in agreement with those reviewed by Majzoub *et al*. (2016) [76], who described the formation of different structures like lamellar phase, inverted hexagonal phase, a hexagonal phase and a gyroid cubic phase. In these kinds of structures, a conserved liposome/lipids/DNA ratio should be observed. Considering that polymerization increases membrane stability [47, 48] by forming lipopolymers within the liposome structures [49], the best model that suits our results cannot be easily chosen. In this sense, deeper conformational studies with cryo-electron microscopy or X-ray scattering need to be performed to determine the structure formed by this kind of cationic lipopolymers when interacting with DNA.

### Hemolysis and viability evaluation in L-929 and Vero cell line

Red blood cells are among the first cells that can interact with a drug delivery system after an intravenous inoculation. Thus, we used a model of human red blood cells to determine the hemolytic effect of our formulations, especially when complexed with DNA. None of the formulations, either complexed or not with the plasmid pDsRed, showed significant differences when compared to the control experiment, at 4- or 24-h of incubation. Ishiwata *et al*. (2000) [28] showed that liposomes containing SA usually increase the permeability of the membranes, damaging the cells. Since our study showed no hemolytic effect for DMPC:DC_8,9_PC:SA (1:1:0.2), we suggest that the lipopolymer context can reduce the effect of free SA. In line with this idea, we have previously shown that DMPC:DC_8,9_PC (1:1) does not present hemolytic effect [47] and in this work, we demonstrated that cationic lipid addition does not increase the *ex vivo* toxicity of the formulation.

However, one of the main problems associated with the use of cationic lipids is their usual cytotoxicity [77]. Thus, the cell lines L929 and Vero were used as a model to test the toxicity of cationic lipopolymers, either complexed or not with plasmid DNA. The cytotoxic effect, studied as metabolic activity, was dependent on the formulation, cell line, lipid concentration and presence of DNA, as detailed in the results section. The DMPC:DC_8,9_PC:DOTAP (1:1:0.2) formulation was the least cytotoxic. A slight tendency to increasing cytotoxicity was observed with the plasmid DNA addition for the DMPC:DC_8.9_PC:SA (1:1:0.2) formulation. At the 16:1 ratio used, not all plasmid DNA was complexed and could lead to the formation of large aggregates, as proposed by Kwon *et al*. (2008) [78]. These aggregates could precipitate on the cells in culture, affecting their viability. However, the toxicity observed was lower than that described by Percot *et al*. (2004) [62] for liposomes composed of a cationic cholesterol derivative and DOPE, which at a concentration of 100 μM reduced the cell viability of B16 F10 cells by 40% when not complexed with DNA. Furthermore, cytotoxicity is dependent on the cell line used and extrapolations between different cell lines and formulations are not convenient [79]. Moreover, as the proper doses for optimal transfection are unknown at this time, a potential cytotoxic effect should not be underestimated at those concentrations.

## Conclusions, perspectives and future applications

As mentioned previously, the mixture of the polymerizable diacetylenic lipid DC_8.9_PC and the lipid DMPC (1:1) has the ability to deliver plasmid DNA and transfect cells in mice after being intratracheally administered. Taking into account this fact, we evaluated two methodologies to incorporate different cationic lipids in this polymer in order to increase the interaction with the nucleic acids. From the point of view of the size and stability of the system, the best results for cationic lipid incorporation were obtained using the "copolymerization" methodology. Furthermore, we showed that the interaction with DNA, its protection, and cytotoxicity is dependent on the cationic lipid used within the lipopolymer. Particularly, the DMPC:DC_8.9_PC:DOTAP (1:1:0.2) formulation was the most efficient in complexing and protecting DNA, forming highly stable complexes resistant to serum DNAs and presenting very low cytotoxicity.

We believe that these cationic lipopolymers have a great potential to be used for *in vivo* applications due to their stability, their ability to protect DNA and their reduced cytotoxicity. Furthermore, the obtained lipopolymers could be used in the context of other techniques intended to control and improve multigene delivery as with the layer-by-layer technique reported by Bishop *et al*. (2016) [80]. Moreover, it would be very interesting to evaluate the performance of these kinds of cationic lipopolymers to interact and protect dsRNA, siRNA or other negatively charged molecules intended to be delivered *in vivo*.

Whether designing a cationic lipopolymer for DNA or RNA delivery, the type of cationic lipid, helper lipid or other variables inherent to the formulation itself must be optimized. This optimization has to consider the particular application for which it is being designed, the correct delivery to the expected tissue and the interaction with the target cell. Also, it has to be taken into account the final destination of the delivered molecule in that cell, as it can be the cytoplasm or the nucleus for the delivery of RNA or DNA, respectively.

Finally, our work highlights the advantage of using cationic lipopolymers based on diacetylenic lipids when designing novel non-viral carriers for use in *in vivo* gene therapy.
